# Carriage of upper respiratory tract pathogens in rural communities of Sarawak, Malaysian Borneo

**DOI:** 10.1186/s41479-021-00084-9

**Published:** 2021-04-25

**Authors:** Denise E. Morris, Hannah McNeil, Rebecca E. Hocknell, Rebecca Anderson, Andrew C. Tuck, Serena Tricarico, Mohd Nor Norazmi, Victor Lim, Tan Cheng Siang, Patricia Kim Chooi Lim, Chong Chun Wie, David W. Cleary, Ivan Kok Seng Yap, Stuart C. Clarke, on behalf of the MYCarriage group, Koh Kwee Choy, Stuart C. Clarke, David W. Cleary, C. Patrick Doncaster, Saul N. Faust, Eddy Cheah Seong Guan, Wong Chin Hoong, Aziah Ismail, Johanna M. C. Jefferies, Alex R. Kraaijeveld, Victor Lim, Nuala McGrath, Michael Moore, Marie-Louise Newell, Mohd Nor Norazmi, Paul Roderick, Ivan Yap Kok Seng, Cheng Siang Tan, Jeremy S. Webb, Chong Chun Wie, Ho Ming Yuen

**Affiliations:** 1 Faculty of Medicine and Institute for Life Sciences, University of Southampton, Southampton, UK; 2 School of Health Sciences, Universiti Sains Malaysia, Kubang Kerian, Kelantan Malaysia; 3 School of Medicine, International Medical University, Kuala Lumpur, Malaysia; 4 Faculty of Medicine and Health Sciences, Universiti Malaysia Sarawak, Kota Samarahan, Sarawak Malaysia; 5 Institute for Research, Development and Innovation, International Medical University, Kuala Lumpur, Malaysia; 6 School of Pharmacy, Monash University Malaysia, Bandar Sunway, Selangor Malaysia; 7 NIHR Southampton Biomedical Research Centre, University Hospital Southampton Foundation NHS Trust, Southampton, UK; 8 Sarawak Research and Development Council, Kuching, Sarawak Malaysia; 9 Global Health Research Institute, University of Southampton, Southampton, UK

**Keywords:** Carriage, Pneumonia, Malaysia, Pathogen, Respiratory, AMR

## Abstract

**Introduction:**

Pneumonia is a leading cause of death in Malaysia. Whilst many studies have reported the aetiology of pneumonia in Western countries, the epidemiology of pneumonia in Malaysia remains poorly understood. As carriage is a prerequisite for disease, we sought to improve our understanding of the carriage and antimicrobial resistance (AMR) of respiratory tract pathogens in Malaysia. The rural communities of Sarawak are an understudied part of the Malaysian population and were the focus of this study, allowing us to gain a better understanding of bacterial epidemiology in this population.

**Methods:**

A population-based survey of bacterial carriage was undertaken in participants of all ages from rural communities in Sarawak, Malaysia. Nasopharyngeal, nasal, mouth and oropharyngeal swabs were taken. Bacteria were isolated from each swab and identified by culture-based methods and antimicrobial susceptibility testing conducted by disk diffusion or E test.

**Results:**

140 participants were recruited from five rural communities. *Klebsiella pneumoniae* was most commonly isolated from participants (30.0%), followed by *Staphylococcus aureus* (20.7%), *Streptococcus pneumoniae* (10.7%), *Haemophilus influenzae* (9.3%), *Moraxella catarrhalis* (6.4%), *Pseudomonas aeruginosa* (6.4%) and *Neisseria meningitidis* (5.0%). Of the 21 *S. pneumoniae* isolated, 33.3 and 14.3% were serotypes included in the 13 valent PCV (PCV13) and 10 valent PCV (PCV10) respectively. 33.8% of all species were resistant to at least one antibiotic, however all bacterial species except *S. pneumoniae* were susceptible to at least one type of antibiotic.

**Conclusion:**

To our knowledge, this is the first bacterial carriage study undertaken in East Malaysia. We provide valuable and timely data regarding the epidemiology and AMR of respiratory pathogens commonly associated with pneumonia. Further surveillance in Malaysia is necessary to monitor changes in the carriage prevalence of upper respiratory tract pathogens and the emergence of AMR, particularly as PCV is added to the National Immunisation Programme (NIP).

**Supplementary Information:**

The online version contains supplementary material available at 10.1186/s41479-021-00084-9.

## Highlights

- Bacterial carriage in the upper respiratory tract in Malaysia is poorly understood.
- *Klebsiella pneumoniae* was the most prevalent bacteria isolated.
- 33.8% of isolates were resistant to at least one antibiotic.

## Introduction

Lower respiratory tract infections, including pneumonia, are the sixth leading cause of death across all ages globally, and the primary cause of mortality in children aged 4 years and under [1]. In Malaysia, pneumonia was the second highest cause of death across all ages in 2017 [2]. Community acquired pneumonia (CAP) is associated with a 4.2% fatality rate [3], which is lower than rates published in Western countries (7.7–12%) [4, 5]. This is possibly due to less-stringent reporting in Malaysia and therefore underestimates of mortality.

Whilst many studies define the aetiology of pneumonia in Western countries, its epidemiology in Malaysia is less understood. Globally *S. pneumoniae* is reported as the most common cause of bacterial pneumonia [6]. Pneumococcal conjugate vaccines (PCVs) offer protection against serotypes of *S. pneumoniae* most commonly associated with disease or antimicrobial resistance (AMR), and are included in the national immunisation programmes (NIPs) of 144 countries worldwide [7]. As of June 2020, Malaysia, Thailand, Sri Lanka, China and Vietnam were yet to include PCV in their NIPs [8]; however the Malaysian government has announced its intention to implement PCV as part of their NIP from late 2020 [9]. PCV vaccine types (VT) 19F, 14, 6B, 1 and 19A are a major cause of invasive pneumococcal disease (IPD) in Malaysia, whilst 19F, 23F, 14, 6B, 1 and 3 are the most prevalent causes in South East Asia [10], therefore PCV implementation is of likely benefit for the reduction of IPD and other diseases including pneumococcal pneumonia.

In addition to *S. pneumoniae*, multi-drug resistant ‘ESKAPE’ pathogens such as *K. pneumoniae* have been reported as common causative organisms of CAP [11, 12]. In contrast to Western countries, studies from Asia suggest *K. pneumoniae* is a more prominent cause of pneumonia than *S. pneumoniae* and *H. influenzae*, [13–15] with data from Malaysia suggesting *K. pneumoniae* to be the most prominent cause [16]. Like *S. pneumoniae*, the World Health Organization considers *K. pneumoniae* a priority AMR pathogen in need of control. Prevention strategies however are hindered by its high level of strain diversity [17]. *K. pneumoniae* is therefore a pathogen of concern. Whilst there is currently no vaccine for *K. pneumoniae*, disease due to *H. influenzae* type b (Hib) has been successfully reduced through the implementation of the Hib vaccine. Despite Hib vaccine implementation and high coverage in Malaysia [18], *H. influenzae* (including Hib) is still a major source of disease such as pneumonia and is a main cause of meningitis [16, 19].

As carriage is a prerequisite for disease [20], we sought to improve our understanding of carriage prevalence and AMR profiles of common upper respiratory tract (URT) pathogens in Malaysia, particularly those associated with pneumonia. This was done by undertaking a population-based carriage study. In particular, we hoped to investigate an understudied part of the Malaysian population (rural communities of Sarawak).

## Methods

### Study site

Sarawak, Sabah and Labuan comprise East Malaysia; also known as Malaysian Borneo due to its location on the island of Borneo. Sarawak is the largest state in Malaysia. The population consists of more than 40 ethnic subgroups; such as Malay, Malaysian Chinese and numerous native communities (commonly referred to as Dayak) comprising Orang Ulu (including Penan, Kelabit and Kenyah), Iban, Bidayuh, Melanau, Tagalm, Punan Bah, Kedayan and Suluk [21]. Many native communities live according to tribal traditions, residing in villages consisting of longhouses, with farming a common occupation. Such communities can be isolated due to their location, restricting access to modern medicines and healthcare services. However, it is becoming increasingly common for some residents to commute, with some staying in larger cities during the working week or longer, for work and study. This offers improved access to healthcare. Furthermore, governmental health initiatives such as a monthly flying doctor and Komuniti Sihat Pembina Negara (KOSPEN) improve public health in such communities by increasing access to medical professionals and raising awareness through the use of community health volunteers.

**Fig. 1:**
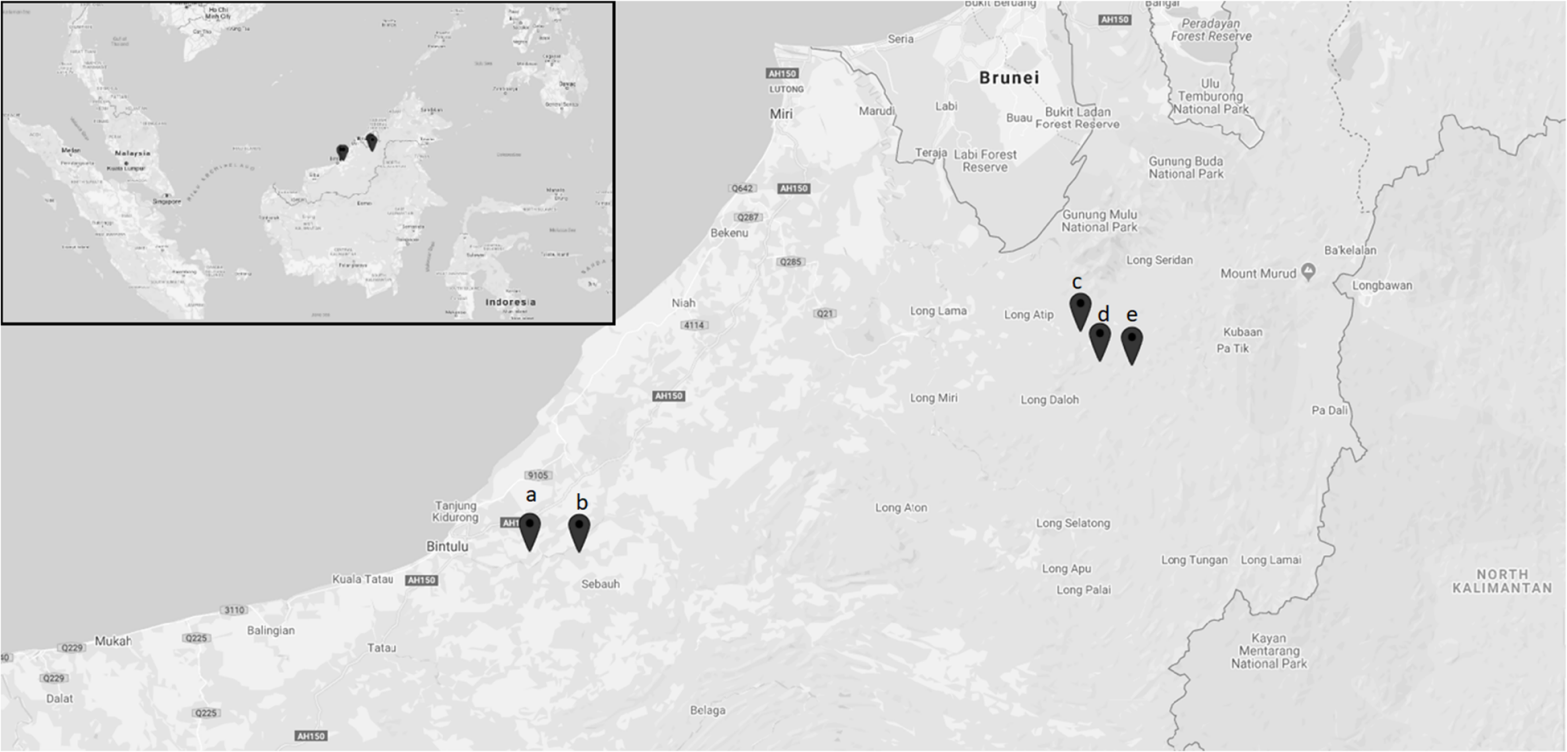
Map of recruitment sites.© Copyright 2020 | Multiplottr.com | All Rights Reserved. **a** Rumah Bana, Sebauh, Bintulu, Sarawak - GPS co-ordinate 3.142234, 113.279135; 43 participants recruited (all Iban). **b** Rumah Numpang, Sebauh, Bintulu, Sarawak - GPS co-ordinate 3.139950, 113.418036; 41 participant recruited (39 Iban, 1 unknown, 1 Penan). **c** Long Kerangan, Ulu Baram, Miri, Sarawak - GPS co-ordinate 3.766312, 114.841122; 19 participants recruited (all Penan). **d** Ba Marong, Ulu Baram, Miri, Sarawak - GPS co-ordinate 3.680444, 115.014777; 13 participant recruited (11 Penan, 1 Kelabit, 1 Kenyah). **e** Long Nen, Ulu Baram, Miri, Sarawak - GPS co-ordinate 3.679871, 114.897062; 24 participants recruited (all Penan)

Five rural communities were sampled (Fig. 1); two were longhouse communities (Rumah Numpang and Rumah Bana) located in Sebauh, Bintulu, Sarawak. Although only 30-40 km from Bintulu town, travel to Rumah Numpang required a 20–30 min longboat ride up the Sungai Pandan and Sungai Sujan rivers to reach an isolated community within a clearing in forest land. Rumah Bana, an affluent community, was less isolated located 20-30 km from Bintulu town off the main road from Sebauh town. The remaining communities (Long Kerangan, Ba Marong and Long Nen) were very isolated, located in Ulu Baram, Miri, Sarawak. Long Kerangan was a highly deprived longhouse community, being isolated and impoverished, located 80 km from Long Lama (the nearest town and healthcare provider) in the middle of forest land. Ba Marong was an even more isolated longhouse community, located in the middle of dense forest 157 km from Long Lama. Travel to this site required trucks with four-wheel drive due to the tough terrain and lack of roads. Long Nen, a village rather than a longhouse community, was situated within dense forest 99 km from Long Lama. The rural communities sampled as part of this study encompassed different tribal lifestyles and included people of Iban, Kelabit, Kenyah and Penan ethnicity.

### Recruitment

A population-based survey of bacterial carriage was undertaken in participants of all ages from five rural communities in Sarawak, Malaysia during April 2016. Rural communities were identified by local community health workers and charities. Prior to each survey, local researchers sent a letter to/approached each longhouse elder/community lead to provide a study overview and seek approval for the team to visit. Researchers visited communities whose elder/lead gave approval and anyone present at each location were approached and invited to participate. Translators and community health workers facilitated recruitment and sampling.

### Sample collection and transport

Bacterial carriage of the URT was sampled by taking whole mouth, nasal, oropharyngeal (OP) and nasopharyngeal (NP) swabs from each participant. Paediatric NP samples were taken using rayon tipped Transwab® Pernasal Amies with charcoal (Medical Wire and Equipment, Corsham, UK). Adult NP samples and all nasal and OP samples were taken using the appropriate viscose tipped sterile Amies swabs with charcoal (Deltalab, Chalgrove, UK). After collection, swabs were chilled where possible before being transported by World Courier at ambient temperature to the University of Southampton, UK. Transportation took 3–5 days.

### Questionnaire

Participants were asked to complete a questionnaire requesting basic demographic data. Parents/guardians of child participants were responsible for the completion of questionnaires.

### Assessment of bacterial carriage

Upon arrival at the laboratory, each swab was immersed and vortexed in skim milk, tryptone, glucose and glycerine (STGG) storage media. For each swab, 10 μL was plated onto Columbia blood agar with horse blood (CBA, Oxoid, UK), Columbia blood agar with chocolated horse blood (CHOC, Oxoid, UK), Columbia Blood Agar with Colisitin and Naladixic Acid (CNA, Oxoid, UK), Columbia Agar with Chocolated Horse Blood and Bacitracin (BACH, Oxoid, UK) and Lysed GC Selective Agar (GC, Oxoid, UK). Plates were incubated for 24–48 h at 37 °C in 5% CO_2_ and examined for the presence of *Moraxella catarrhalis, S. pneumoniae, H. influenzae, Neisseria meningitidis, Staphylococcus aureus* and *K. pneumoniae*. For the isolation of *Pseudomonas aeruginosa*, 10 μL was plated onto *Pseudomonas* CFC Selective agar (CFC, Oxoid, UK) and incubated for 24–48 h at 37 °C. Primary identification of all bacterial species was by colonial morphology. Bacteria of interest were then confirmed (Supplementary Methods 1) and isolates frozen at − 80 °C in STGG.

### Pneumococcal serotyping

*S. pneumoniae* were serotyped by slide agglutination reactions using a Neufeld *S. pneumoniae* antisera kit (Statens Serum Institute, Copenhagen, Denmark).

### Antibiotic susceptibility

Bacterial isolates were phenotypically tested for antibiotic resistance using MRSA agar and antibiotic discs and/or minimum inhibitory concentration (MIC) strips, in accordance to EUCAST (Supplementary Methods 1).

### Carriage prevalence

Prevalence of carriage was calculated as a percentage by dividing the number of bacteria isolated by the total number of participants swabbed. This was done for each bacterial species according to anatomical sample site and age group. True carriage, defined as identification of a bacterial species in any specimen from an individual participant regardless of the site or number of carriage sites per person, was also determined.

### Statistical analysis

The Fisher’s exact test was used to determine the significance of association between an independent and dependent variable (e.g. bacterial carriage and age group). A *p-value* of 0.05 was used as a significant threshold. All data analysis was done using GraphPad Prism version 7.03 for Windows (GraphPad Software, San Diego, CA, USA).

## Results

In total 140 participants were recruited from five communities. Grouped by age, 8.6% (*n* = 12) of participants were 0–4 years, 20.0% (*n* = 28) were 5–17 years, 70.7% (*n* = 99) were 18 years and above and 0.7% (*n* = 1) were of unknown age. 44.3% (*n* = 62) of participants were male and 54.3% (*n* = 76) were female, whilst 1.4% (*n* = 2) of participants did not provide their gender. Regarding ethnicity, 58.6% (*n* = 82) of participants were Iban, 0.7% (*n* = 1) Kelabit, 0.7% (*n* = 1) Kenyah, 39.3% (*n* = 55) Penan participants and 0.7% (*n* = 1) did not provide ethnicity. All 140 participants provided mouth and nasal swab samples; 93.6% (*n* = 131) provided NP and 92.1% (*n* = 129) provided OP swab samples.

**Fig. 2:**
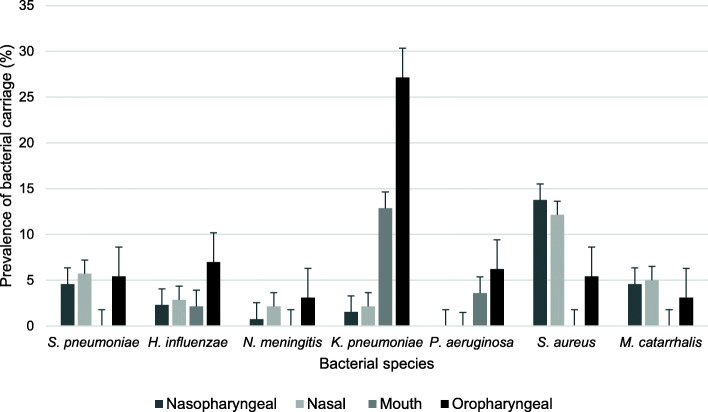
Prevalence of bacterial carriage at each anatomical sample site. Prevalence bar plot showing bacterial carriage by anatomical sample site in participants recruited from rural communities in Sarawak, Malaysia in April 2016. Error bars show standard error

**Fig. 3:**
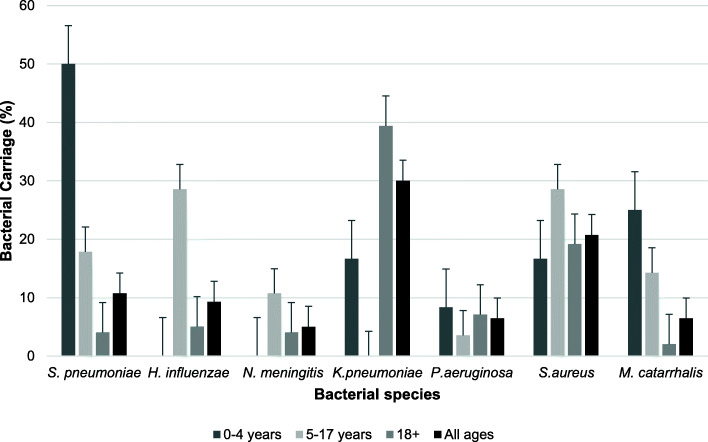
True bacterial carriage of each bacteria by age group. Prevalence bar plot showing true bacterial carriage prevalence by age in participants recruited from rural communities in Sarawak, Malaysia in April 2016. True carriage is defined as at least one carriage event observed regardless of the site of isolation or the number of sites from which the bacterium was recovered. Error bars show standard error

Prevalence of bacterial carriage was determined by anatomical sample site (Fig. 2). The organism most commonly isolated from the oropharynx and mouth was *K. pneumoniae* with a carriage prevalence of 27.1% (95CI: 19.7–35.74%; *n* = 35) and 12.9% (95CI: 7.8–19.6%; *n* = 18) respectively. For the nasopharynx and nose however, *S. aureus* was most prevalent at 13.7% (95CI: 8.4–20.8%; *n* = 18) and 12.1% (95CI: 7.2–18.7%; *n* = 17) respectively. All other species of interest showed much lower carriage levels at each of the anatomical sites sampled. True carriage was also determined (Fig. 3). *K. pneumoniae* was the most prevalent bacteria with a true carriage prevalence of 30.0% (95CI: 22.6–38.3%; *n* = 42), followed by *S. aureus* at 20.7% (95CI: 14.3–28.4%; *n* = 29), *S. pneumoniae* at 10.7% (95CI: 6.1–17.1%; *n* = 15), *H. influenzae* at 9.3% (95CI: 5.0–15.4%; *n* = 13), *M. catarrhalis* at 6.4% (95CI: 3.0–11.9%; *n* = 9), *P. aeruginosa* at 6.4% (95CI: 3.0–11.9%; n = 9) and *N. meningitidis* at 5.0% (95CI: 2.0–10.0%; *n* = 7).

When age group is considered, *S. pneumoniae* were carried significantly more in children aged 0–4 years (*P < 0.05*) (Fig. 3), and there were significantly more *H. influenzae* isolated from those aged 5–17 years than children under 5 years and adults (*P < 0.05*). *K. pneumoniae* were carried significantly more in adults than children (*P < 0.05*).

**Fig. 4:**
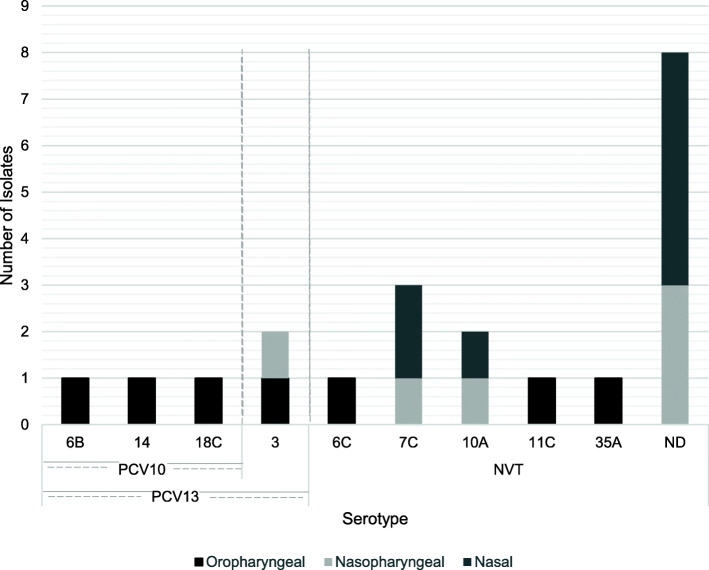
Serotypes of the *S. pneumoniae* isolated. Bar plot showing the observed numbers for each serotype identified for the 21 pneumococcal isolates collected from the oropharynx, nasopharynx, nose and mouth of participants recruited from rural communities in Sarawak, Malaysia in April 2016. ND: Not determined. PCV: pneumococcal conjugate vaccine, NVT: Non-vaccine type. Both serotype 3 *S. pneumoniae* were isolated from the same participant, one from the nasopharynx the other from the oropharynx. Two of the serotype 7C *S. pneumoniae* were isolated from the same participant, one from the nasopharynx the other from the nose. Both serotype 10A *S. pneumoniae* were isolated from the same participant, one from the nasopharynx the other from the nose. 6 of the un-typed *S. pneumoniae* were isolated from 3 participants with each having *S. pneumoniae* isolated from the nasopharynx and nose

Of the 21 pneumococci isolated, 33.3% (*n =* 7) were PCV13 VT (6B, 14, 18C and 3), 14.3% (*n* = 3) were PCV10 VT (6B, 14 and 18C) and 28.6% (*n* = 6) were non-vaccine types (NVT) (6C, 7C, 10A, 11C and 35A) (Fig. 4). The final 38.1% (*n* = 8) could not be serotyped.

**Fig. 5:**
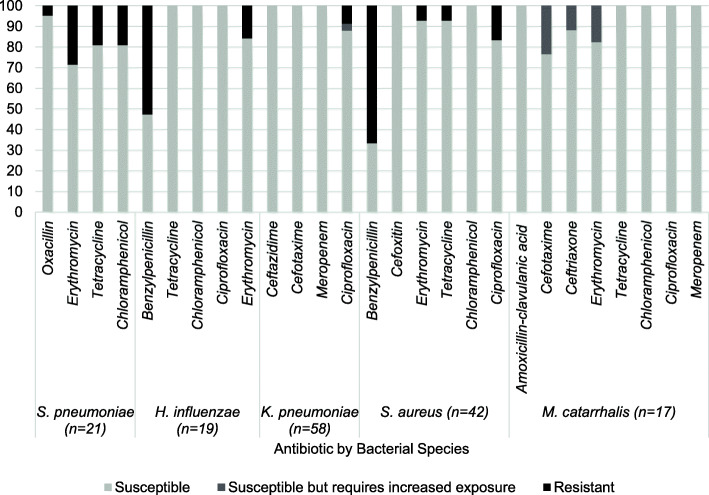
Antimicrobial resistance of bacteria isolated. Percentage bar plot showing the AMR of all 157 *S. pneumoniae*, *H. influenzae*, *K. pneumoniae*, *S. aureus* and M. catarrhalis isolates collected from residents of rural communities in Sarawak, Malaysia in April 2016. Antibiotic discs and MIC strips were used and EUCAST breakpoints were used to assess susceptibility and resistance

All *S. aureus* isolates were identified as methicillin sensitive. When assessing antibiotic susceptibility of bacterial isolates using antibiotic discs and MIC strips, susceptibility ranged from 33.3–100% dependant on species and antibiotic (Fig. 5). With the exception of *S. pneumoniae*, all isolates of each bacterial species were susceptible to at least one type of antibiotic (e.g. all *H. influenzae* isolates were susceptible to ciprofloxacin), whilst all bacterial isolates, no matter the species, were susceptible to one or more of the antibiotics they were tested against. Of the 21 *S. pneumoniae* isolated, 47.6% (*n* = 10) were resistant to at least one antibiotic and 23.8% (*n* = 5) were resistant to two antibiotics. None were resistant to three or more antibiotics. Of the 19 *H. influenzae* isolated, 52.6% (*n =* 10) were resistant to at least one antibiotic and none were resistant to two or more antibiotics. Of the 42 *S. aureus* isolated, 66.7% (*n* = 28) were resistant to at least one antibiotic, 28.6% (*n* = 12) were resistant to at least two antibiotics and 2.3% (*n =* 1) were resistant to three antibiotics. Of the 17 *M. catarrhalis* isolated none were resistant to any antibiotic tested. Of the 58 *K. pneumoniae* isolated, 8.6% (*n =* 5) were resistant to at least one antibiotic and none were resistant to two or more antibiotics. Overall, 33.8% (*n* = 53) of all bacteria isolated and tested (*n* = 157) were resistant to at least one antibiotic, 10.8% (*n* = 17) to at least two antibiotics and 0.6% (*n =* 1) to three antibiotics. The highest prevalence of resistance was observed for *S. aureus* with 66.7% resistant to benzylpenicillin. This was followed by *H. influenzae* resistance to benzylpenicillin (52.6%) and *S. pneumoniae* to erythromycin (28.6%).

## Discussion

This study aimed to address the paucity of published epidemiological data in Malaysia, by increasing our knowledge of the carriage and AMR profiles of common URT pathogens. It has provided important data regarding bacterial epidemiology in an understudied part of the Malaysian population. As well as an invaluable perspective as to the potential challenges of infection and AMR control in such populations, which are isolated to varying degrees from the wider Malaysian population and/or are socio-economically distinct from those living in large towns or Peninsular Malaysia due to their rural or longhouse lifestyles. In Malaysia the epidemiology of URT pathobionts is poorly understood and would therefore benefit from further large-scale population-based studies to inform vaccine policy, to monitor the effects of vaccine implementation and provide an understanding of the prevalence of AMR to inform treatment of infection [22].

This study provided a particular opportunity to investigate ESKAPE pathogens such as *K. pneumoniae*, whose morbidity and mortality has been linked to an increased emergence of AMR [12]. Further surveillance is required to monitor changes in carriage prevalence, the emergence of new serotypes and AMR [23]. We found *K. pneumoniae* to be carried significantly more in adults, which is comparable to findings from Indonesia [24], however carriage of *K. pneumoniae* here was more than double that previously reported in Indonesia, Vietnam, Brazil and Minnesota, USA (between 3 and 15%) [24–27]. This is noteworthy because carriage is a risk factor for infection and *K. pneumoniae* is a common cause of CAP and serious sequela such as sepsis and meningitis [25, 28]. *K. pneumoniae* is of increasing concern *due to* the emergence of multidrug-resistant strains associated with hospital outbreaks and severe community-acquired infections. Last-line defense antibiotics are quickly becoming ineffective [29]. Despite this, isolates obtained in this study showed low prevalence of AMR, and none were multidrug resistant. Whilst only four antibiotics were tested, those chosen are the most clinically relevant based on prescription guidelines in Malaysia. Even though both interest and published clinical data for *K. pneumoniae* has increased, little has been published on the AMR of carriage isolates. A previous study found 29% of URT carriage isolates to have resistance to two of the antibiotics tested, with 100% of isolates being resistant to ampicillin [30]. Despite *K. pneumoniae* being considered an extended-spectrum beta-lactamase (ESBL)-producing bacteria [31], high levels of antibiotic susceptibility were found, even to β-lactam antibiotics [30], which is consistent with the findings of this study.

Carriage of *S. aureus* was consistent with the 23.4% carriage prevalence previously found in Peninsular Malaysia [32]. Due to a lack of data, the carriage prevalence of *S. pneumoniae, H. influenzae* and *M. catarrhalis* in Malaysia is unclear. Carriage data from young children in Indonesia has shown 49.5, 27.5 and 42.7% prevalence for *S. pneumoniae, H. influenzae and M. catarrhalis* respectively [33]. Due to the small sample size of this age group in our study, the carriage prevalence of these bacteria is imprecise; 50% (95CI: 21.8–78.2%), 0% (95CI: 0.0–26.5%), and 25% (95CI: 0.60–49.4%) for *S. pneumoniae, H. influenzae and M. catarrhalis* respectively. Further research would be useful in all age groups.

Whilst our study found 33.3% of carried *S. pneumoniae* serotypes to be PCV13 VT and 14.3% PCV10 VT, studies from Peninsula Malaysia showed a higher proportion of *S. pneumoniae* VT carriage, with up to 69.5% being PCV13 VT and 40.6% being PCV10 and PCV7 VT [34, 35]. Certainly, VT *S. pneumoniae* cause the majority of pneumococcal disease in Peninsular Malaysia with 66.3% of RTI in children being caused by PCV13 VT and 60% by PCV10 VT [36]. As PCV13 serotypes 1, 6B, 14, 19F, 19A are commonly associated with disease in Malaysia [10, 37], the carriage of 6B and 14 (which was in children) here is of interest for public health, particularly as these serotypes are included in both PCV10 and PCV13. However, since only 21 isolates of *S. pneumoniae* were recovered, limited conclusions can be drawn, especially as many remained un-typed. However, as PCV coverage of invasive isolates in Malaysia has been reported to be as high 88.2% for PCV13 and 64.1% for PCV10 the widespread uptake of PCV as part of the planned NIP update has a high potential to reduce disease [10, 38].

AMR is an increasing issue across Asia; multidrug resistant *S. pneumoniae* and *K. pneumoniae* are of particular concern [17, 36, 39, 40]. In Malaysia, antibiotics are commonly prescribed for the treatment of URT infections, although inappropriate antibiotic choice and dosage have been shown in up to 40% of cases. This is important, as the misuse of antibiotics is a significant selective force for the development of AMR. Erythromycin and penicillin are the most commonly prescribed and the most commonly over or incorrectly prescribed [41]. They were also the antibiotics shown to have the highest levels of resistance in this study. Furthermore, data suggests 55% of Malaysians discontinue their course of antibiotics when symptoms disappear, rather than completing the full course as prescribed. Such misuse is a significant risk factor for the development of AMR and is most common in community rather than hospital settings [42]. To reduce antibiotic misuse, education on the risks of AMR is being provided in accordance to the Malaysian Action Plan on Antimicrobial Resistance (MyAP-AMR) [23, 42].

Our AMR data contrasts with previous studies in Peninsular Malaysia; here 66.7% (*n* = 28) of *S. aureus* were resistant to benzylpenicillin compared to 100% seen previously [43]. Erythromycin resistance in *S. pneumoniae* was comparable to that from disease in Peninsular Malaysia study (28.6% versus 35.8%) whilst tetracycline resistance was lower (19.1% versus 38.7%) [44].

The main limitation of this study was that limited recruitment, especially in children, meant some carriage prevalence had wide confidence intervals. Low child recruitment was exacerbated by not all children providing NP or OP swabs. Of the 40 children recruited, only 32 provided NP swabs and 29 provided OP swabs; all provided nose and mouth swabs. Several factors accounted for low recruitment. In Rumah Numpang and Rumah Bana a high proportion of the community (particularly males) live away for work/study and only return on alternate weekends, whilst others live away for months at a time. Furthermore, some residents avoided areas that the study team were authorised to recruit from to avoid interaction with researchers/taking part in the study (Long Nen), whilst other sites were very small communities (Ba Marong comprised only two extended families). This study does however inform the feasibility of undertaking such research. Ways to improve recruitment such as the need to visit more sites or make repeat visits to sites have been identified. Although difficult for some communities, particularly those most isolated, authors would recommend increased community engagement prior to the research visit. Despite the University having links to the some of the communities and engaging with the leaders prior to the study, increased engagement may enable increased trust from community residents, reducing avoidance of researchers. It may also allow researcher to have better awareness of the best times to visit when most residents are likely to be present.

Limitations arose during the transportation of swabs, firstly due to temperature fluctuations between sample collection and arrival at UK laboratories because of unrefrigerated transportation conditions. During recruitment and sampling, utilised swabs were stored in insulated boxes with ice to keep temperatures low; however, temperatures reached up to 30 °C by arrival at excursion headquarters. At excursion headquarters and during ground courier transportation, swabs were kept in air-conditioned buildings and vans so temperatures remained below 21 °C. On the flight from Malaysia to the UK, the temperature was much lower (less than 10 °C), whilst ground transportation in the UK likely remained between 2 and 15 °C. Despite this, the temperature range of swab storage was compliant with the quality control conditions tested under Clinical Laboratory Standards Institute (CLSI) Approved Standard M40-A2 [45]. All swabs used in this study were CLSI Approved Standard M40-A2, which ensures bacterial numbers will not reduce by more than 3 log10 under at refrigeration and ambient temperature (2–30 °C) or increase by more than 1 log10 at refrigeration temperature, during a 48 h holding period. The second limitation regarding swab transportation, was the time between sampling and the swabs being processed in the laboratory. As transportation took 3–5 days, there is the potential that the carriage prevelances seen here may be underestimates due to some bacteria not surviving transportation. Certainly, transport conditions would have reduced bacterial viability, and may have affected some less resilient species more than others. Future studies would benefit from the use of dry shippers, which would combat the issue of temperature fluctuations and transportation delays. Furthermore, it may be of benefit to immediately store swab samples in STGG whilst in the field, rather than waiting until swabs are received by the laboratory. This would further facilitate freezing/use of dry shippers prior to transportation.

The need to transport swabs to UK based laboratories resulted from a lack of capacity at the time for microbial analyses at local collaborating institutions. This advocates building research and laboratory capacity in Malaysia.

Since whole genome sequencing was not possible in this study, many (38.1%, *n* = 8) *S. pneumoniae* remained un-typed; it is unclear whether they were non-typeable *S. pneumoniae* (lacking a capsule), were novel serotypes, or typical serotypes that were unable to be typed with the agglutination methodology used. Such data would have contributed to further understanding the potential benefit of widespread uptake in PCV in Malaysia and thus will be included in future research.

## Conclusion

This study reports novel data on an understudied part of the Malaysian population, providing important insights into the epidemiology and AMR profiles of common respiratory pathogens associated with pneumonia.

## Supplementary Information

**Additional file 1.** Supplementary Methods 1.

## Data Availability

The datasets used and/or analysed during the current study are available from the corresponding author on reasonable request.
