# Supplementary material for: Carriage of upper respiratory tract pathogens in rural communities of Sarawak, Malaysian Borneo
Source: Pneumonia (Nathan). 2021 Apr 25;13:6. doi: 10.1186/s41479-021-00084-9 (PMC8070298; doi:10.1186/s41479-021-00084-9)
Supplement: Supplementary file 1 — Additional file 1. Supplementary Methods 1. [file 41479_2021_84_MOESM1_ESM.docx]

**Supplementary Methods 1**

Bacterial Confirmation

Suspected *S. pneumoniae* were confirmed as Gram-positive diplococci and susceptible to optochin (Oxoid, Basingstoke, UK) [2].

Suspected *H. influenzae* were confirmed as oxidase positive coccobacilli. Final confirmation was undertaken by X and V testing. Isolates which grew around X+V discs (Oxoid, Oxford, UK), but not the individual X or V discs (Oxoid, Oxford, UK) were verified as *H. influenzae* [3].

Suspected *M. catarrhalis* were verified as oxidase, tributyrin and DNAse positive diplococci [4]. Tests were done using oxidase strips (Oxoid, Basingstoke, UK), tributyrin strips (Sigma- Aldrich, UK) and DNAse/methyl green plates (VWR, UK) in accordance with manufacturer’s instructions.

Suspected *N. meningitidis* were confirmed as Gram-negative cocci and oxidase positive [5]. Final confirmation was achieved by performing API NH testing (Biomerieux, UK), in accordance with manufacturer’s instructions.

Suspected *S. aureus* were confirmed as Gram-positive cocci and coagulase positive [6]. Coagulase activity was assessed for each isolate using a Pastorex Staph Plus Kit (Bio-Rad, UK).

Suspected *P. aeruginosa* were confirmed as Gram-negative rods and oxidase positive [7]. Final confirmation was achieved by performing API 20E testing, in accordance with manufacturer’s instructions.

Suspected *K. pneumoniae* were sub-cultured onto CLED agar (Oxoid, UK) and confirmed via matrix-assisted laser desorption ionization time-of-flight mass spectrometry (MALDI-TOF).

Antibiotic Susceptibility

Confirmed *S. aureus* isolates were cultured to (CBA, Oxoid, UK) and incubated at 37^°^C overnight. A single colony from the CBA plate for each isolate was then cultured directly to Brilliance MRSA 2 agar (Oxoid, UK) and incubated for 18-24 hours, at 37^°^C to confirm Methicillin-resistant or Methicillin-sensitive (MRSA/MSSA) status. A methicillin resistant strain (NCTC 12493) and a sensitive strain (ATCC 29213) were used as positive and negative controls respectively.

Bacterial isolates were phenotypically tested for antibiotic resistance using antibiotic discs and/or minimum inhibitory concentration (MIC) strips, in accordance to EUCAST. Firstly, 10μL (a suspension of cells in liquid STGG) of each isolate was plated onto CBA (Oxoid, UK) or CHOC agar (Oxoid, UK). *M. catarrhalis*, *S. pneumoniae, S. aureus* and *K. pneumoniae* isolates were plated on CBA, whilst *H. influenzae* isolates were plated onto CHOC agar. Plates were incubated for 24 hours at 37^°^C in 5% CO_2_. Pure colonies were added to 1ml of saline to get an inoculum of 0.5 McFarland. For *M. catarrhalis, S. pneumoniae and H. influenzae*, a sterile swab was used to spread this inoculum over Mueller-Hinton agar + 5% defibrinated horse blood and 20 mg/L β-NAD plates (MHF, Oxoid, UK). Antibiotic discs (Oxoid, UK) (four per plate) or MIC strips (E-tests; Oxoid, UK) (one per plate) were added and plates were incubated at 35 ± 1^°^C in 5% CO_2_ for 18 hours (±2 hours). For *S. aureus* and *K. pneumoniae* a sterile swab was used to spread the inoculum over Mueller-Hinton agar plates (MH, Oxoid, UK). Antibiotic discs (Oxoid, UK) (four per plate) or MIC strips (E-tests; Oxoid, UK) (one per plate) were added and plates were incubated at 35 ± 1^°^C for 18 hours (±2 hours).

*M. catarrhalis* were tested with amoxicillin-clavulanic acid (2-1ug), cefotaxime (5ug), ceftriaxone (30ug), erythromycin (15ug), tetracycline (30ug), chloramphenicol (30ug), ciprofloxacin (5ug) and meropenem (10ug) antibiotic discs. *S. pneumoniae* were tested with oxacillin (1ug), erythromycin (15ug), tetracycline (30ug) and chloramphenicol (30ug) antibiotic discs. *H. influenzae* were tested with benzylpenicillin (1ug), tetracycline (30ug), chloramphenicol (30ug) and ciprofloxacin (5ug) antibiotic discs as well as erythromycin (15ug) MIC strips. *S. aureus* were tested with benzylpenicillin (1ug), erythromycin (15ug), cefoxitin (30ug), tetracycline (30ug), chloramphenicol (30ug) and ciprofloxacin (5ug) antibiotic discs. *K. pneumoniae* were tested with cefotaxime (5ug), ciprofloxacin (5ug), meropenem (10ug) and ceftazidime (10ug) antibiotic discs.

[1] Vincent JR, Ali RM. Managing natural wealth : environment and development in Malaysia. Washington, D.C.; Singapore: RFF Press ; Institute of Southeast ASian Studies (ISEAS); 2005.

[2] Public Health England. Identification of Streptococcus species, Enterococcus species and Morphologically Similar Organisms. 2014.

[3] Public Health England. Identification of Haemophilus species and the HACEK Group of Organisms 2015.

[4] Public Health England. Identification of Moraxella species and Morphologically Similar Organisms. 2015.

[5] Public Health England. Identification of Neisseria species. 2015.

[6] Public Health England. Identification of Staphylococcus species, Micrococcus species and Rothia species. 2014.

[7] Public Health England. Identification of Pseudomonas species and other non glucose fermenters. 2015.
